# Impact of *fibroblast growth factor receptor 1* (*FGFR1*) amplification on the prognosis of breast cancer patients

**DOI:** 10.1007/s10549-020-05865-2

**Published:** 2020-08-27

**Authors:** Ramona Erber, Matthias Rübner, Simon Davenport, Sven Hauke, Matthias W. Beckmann, Arndt Hartmann, Lothar Häberle, Paul Gass, Michael F. Press, Peter A. Fasching

**Affiliations:** 1 Institute of Pathology, Comprehensive Cancer Center EMN, Erlangen University Hospital, Friedrich Alexander University of Erlangen–Nuremberg (FAU), Krankenhausstrasse 8-10, 91054 Erlangen, Germany; 2 Department of Obstetrics and Gynecology, Comprehensive Cancer Center EMN, Erlangen University Hospital, Friedrich Alexander University of Erlangen–Nuremberg (FAU), Erlangen, Germany; 3 Department of Pathology, Norris Comprehensive Cancer Center, University of Southern California, Los Angeles, CA USA; 4 ZytoVision GmbH, Bremerhaven, Germany; 5 Biostatistics Unit, Department of Gynecology and Obstetrics, Erlangen University Hospital, Erlangen, Germany

**Keywords:** Breast cancer, *FGFR1*, *FGFR2*, *FGFR3*, FISH, Amplification, Prognosis

## Abstract

**Purpose:**

Various aberrations in the fibroblast growth factor receptor genes *FGFR1*, *FGFR2*, and *FGFR3* are found in different cancers, including breast cancer (BC). This study analyzed the impact of *FGFR* amplification on the BC prognosis.

**Methods:**

The study included 894 BC patients. The amplification rates of *FGFR1*, *FGFR2,* and *FGFR3* were evaluated on tissue microarrays using fluorescence in situ hybridization (FISH). Associations between these parameters and prognosis were analyzed using multivariate Cox regression analyses.

**Results:**

*FGFR1* FISH was assessable in 503 samples, *FGFR2* FISH in 447, and *FGFR3* FISH in 562. The *FGFR1* amplification rate was 6.6% (*n* = 33). Increased *FGFR2* copy numbers were seen in 0.9% (*n* = 4); only one patient had *FGFR3* amplification (0.2%). Most patients with *FGFR1* amplification had luminal B-like tumors (69.7%, *n* = 23); only 32.6% (*n* = 153) of patients without *FGFR1* amplification had luminal B-like BC. Other patient and tumor characteristics appeared similar between these two groups. Observed outcome differences between BC patients with and without *FGFR1* amplification did not achieve statistical significance; however, there was a trend toward poorer distant metastasis-free survival in BC patients with *FGFR1* amplification (HR = 2.08; 95% CI 0.98 to 4.39, *P* = 0.05).

**Conclusion:**

*FGFR1* amplification occurs most frequently in patients with luminal B-like BC. The study showed a nonsignificant correlation with the prognosis, probably due to the small sample size. Further research is therefore needed to address the role of *FGFR1* amplifications in early BC patients. *FGFR2* and *FGFR3* amplifications are rare in patients with primary BC.

**Electronic supplementary material:**

The online version of this article (10.1007/s10549-020-05865-2) contains supplementary material, which is available to authorized users.

## Introduction

Breast cancer (BC) is the most common malignant tumor in women [17]. Treatment decisions in breast cancer patients are based on tumor predictive markers [estrogen receptor (ER), progesterone receptor (PR), human epidermal growth factor receptor 2 (HER2)], some of which are also prognostic markers (ER, PR, HER2, Ki-67).

The prognostic and predictive values of many different biomarkers in relation to breast cancer have been evaluated in recent years. The discovery of HER2 amplification/overexpression as a therapeutic target and the development of the first anti-HER2 agent, trastuzumab, were pioneering advances [53]. In the era of personalized medicine, more and more genetic aberrations in potentially targetable oncogenic driver genes, such as copy number variations of *CCND1* and *PIK3CA* mutations, are now being investigated [9, 40].

Another promising biomarker in breast cancer is the fibroblast growth factor receptor 1 gene (*FGFR1*, chromosomal region: 8p11.2-p12). It belongs to a family of receptor tyrosine kinases, activated by fibroblast growth factors, that influence the downstream MAPK, PI3K-AKT-mTOR, and STAT pathways. Stimulation of FGFR1 physiologically leads to proliferation, survival, migration, and angiogenesis [14, 59]. Amplification of *FGFR1* is found in several types of cancer (e.g., nonsmall cell lung carcinoma, head and neck tumors, breast cancer, ovarian cancer, bladder cancer, and rhabdomyosarcoma) [10, 11, 22, 38, 52], with a frequency of up to 10% in breast cancer [61]. Chromosomal aberration has been found to be associated with *FGFR1* overexpression, luminal B subtype (16–27%), negative PR expression, and high Ki-67 protein expression [61]. In addition, breast cancer cell lines with *FGFR1* amplification harbor endocrine resistance that can be reversed by RNA silencing, and *FGFR1*-amplified breast cancers have been reported to be associated with a poorer prognosis [13].

In addition to *FGFR1,* fibroblast growth factor receptor 2 (*FGFR2* gene, chromosome 10) and fibroblast growth factor receptor 3 (*FGFR3* gene, chromosome 4) belong to the same family of receptor tyrosine kinases and are linked to breast cancer susceptibility. Single-nucleotide polymorphisms (SNPs) in *FGFR2* locus 10q26 have been reported to have the strongest association with breast cancer risk in genome-wide association studies [12, 15, 28, 35–37]. *FGFR2*-amplified breast cancer was found with frequencies of up to 4.4% [7]. Wein et al. [63] reported the case of a patient with *FGFR2*-amplified metastatic hormone receptor-positive breast cancer, who benefited from therapy with the mTOR inhibitor everolimus and exemestane. The authors also carried out an analysis of the METABRIC (Molecular Taxonomy of Breast Cancer) dataset [9] and found a 1.8% rate of *FGFR2* amplification in breast cancer, associated with a poorer prognosis and resistance to endocrine therapy [63]. *FGFR3* has also been linked to an influence on endocrine resistance [57] and the risk of breast cancer (e.g., via SNPs) [1], but the amplification rate has been reported to be less than 1% [26].

Since fibroblast growth factor receptor (FGFR) alterations are found in a variety of cancers [64], several FGFR inhibitors—both pan-FGFR and also selective FGFR inhibitors—have been developed and tested in clinical trials [30, 62]. The results of these studies will show whether cancer patients are able to benefit from this targeted therapy. However, assuming that there is an association between *FGFR1* amplification and prognostically unfavorable luminal B breast cancer, it may be hypothesized that *FGFR* inhibitors may improve the prognosis, particularly in patients who are suffering from highly proliferative, hormone receptor-positive breast cancer with *FGFR1* amplification.

The aim of this study was to investigate the amplification rates of *FGFR1*, *FGFR2,* and *FGFR3* in patients with breast cancer and their impact on prognosis.

## Materials and methods

### Patient cohort

The Bavarian Breast Cancer Cases and Control (BBCC) study, described in detail elsewhere [16], was a case–control study that initially included 1538 women with breast cancer, who received various treatments in accordance with University Breast Center guidelines at the University Breast Center for Franconia, which is part of the University Hospital Erlangen (Bavaria, Germany). Tumor samples were collected from 1997 to 2007 [65, 66]. Approval for the study was obtained from the local ethics committee at the University of Erlangen (ref. numbers 2700 and 297_17 Bc). The study was conducted in concordance to “Reporting recommendations for tumor marker prognostic studies (REMARK)” [34].

### Collection of clinical and histopathological data

Clinical and follow-up data were obtained from the patients’ records. Data for histopathological parameters—TNM, grading, ER status, PR status, HER2 status, and proliferation rate measured with Ki-67 immunohistochemistry (IHC)—were obtained from the original pathology files. The detailed methods of assessing these parameters have been described elsewhere [16]. Molecular-like breast cancer subtypes were defined as follows:

- Luminal A-like: ER-positive and/or PR-positive, in at least 10% of tumor cell nuclei (through December, 2009) or in at least 1% of tumor cell nuclei (since January, 2010); HER2-negative, Ki-67 < 14%
- Luminal B-like (HER2-negative): ER-positive and/or PR-positive, HER2-negative, Ki-67 ≥ 14% [4];
- HER2-enriched: HER2 + by either immunohistochemistry (IHC 3 +) [45] or fluorescence in situ hybridization (FISH) or both [43, 44, 46].
- Basal-like or triple-negative: ER-negative, PR-negative, and HER2-negative.

### Fluorescence in situ hybridization of *FGFR1, FGFR2,* and *FGFR3*

After tissue microarrays (TMAs) of formalin-fixed, paraffin-embedded tumor tissue had been built [16], fluorescence in situ hybridization (FISH) was performed in accordance with the manufacturer’s recommendations and in-house standards. The FISH probes used were Zyto*Light* SPEC *FGFR1*/*CEN8* Dual Color Probe, Zyto*Light* SPEC *FGFR2*/*CEN10* Dual Color Probe, and Zyto*Light* SPEC *FGFR3*/*4p11* Dual Color Probe (all from ZytoVision GmbH, Bremerhaven, Germany). These each contained a green-labeled probe that targeted the *FGFR* gene locus (*FGFR1*, *FGFR2,* or *FGFR3*) and an orange-labeled probe that targeted the centromeric region of the particular chromosome (*CEN8*, *CEN10*, *4p11*, respectively). For each TMA core, the green signal (*FGFR* gene locus) and orange signal (centromeric region) were counted in 20 tumor nuclei each, and the *FGFR*/*CEN* ratio was calculated. An *FGFR*/*CEN* ratio ≥ 2.0 was defined as amplification of each fibroblast growth factor receptor. To verify the validity of FISH staining positively (amplification) and negatively (no amplification), the following cell lines were used for validation: MDA MB-134, SUM-190, MFM-223, SNU-16, Kato III, HCC-70, MDA MB-361, BT-20, and MCF-7.

In order to rule out intratumoral heterogeneity of FGFR1 amplification, additional *FGFR1* FISH analyses for a subgroup (*n* = 149) of the initial TMA cohort (TMA_1) were done. For the subcohort, FISH analyses were performed using two more TMAs (TMA_2 and TMA_3) that included each one further area of the tumor area unrelated to the tumor spot that was investigated initially in TMA_1. Results were shown with cross tabulations.

### Statistical analysis

Due to small numbers of *FGFR2-*amplified and *FGFR3*-amplified cases, statistical analysis was limited to *FGFR1* gene status. Disease-free survival (DFS) was defined as the time from the date of diagnosis to the earliest date of disease progression (distant metastasis, local recurrence, death from any cause) or the date of censoring. Patients who were lost to follow-up before the maximum observation period of 10 years, or who were disease-free after the maximum observation time, were censored at the last date they were known to be disease-free or at the maximum observation time. Distant metastasis-free survival (DMFS), overall survival (OS), and local recurrence-free survival (LRFS) were defined similarly.

The primary objective was to study the impact of *FGFR1* on DFS. For this purpose, a simple Cox regression analysis with *FGFR1* amplification (yes/no) as predictor was performed in order to obtain an unadjusted hazard ratio (HR) with 95% confidence intervals (CI) and corresponding *P* values. Survival rates were estimated using the Kaplan–Meier product limit method. An adjusted HR for *FGFR1* amplification was estimated using a multiple Cox regression model with *FGFR1* amplification as predictor, along with well-known prognostic characteristics of DFS: age at diagnosis (continuous), body mass index (BMI, continuous), tumor stage (ordinal, T1 to T4), tumor grade (ordinal, 1 to 3), ER status (positive versus negative), PR status (positive versus negative), HER2 status (positive versus negative), and Ki-67 (continuous, 0–100%). Lymph-node stage was incorporated into the model as a stratification factor (N0, N +) rather than a predictor, as the proportional hazards assumption was violated. Patients with missing information on *FGFR1* gene status were excluded. Missing predictor values were imputed, and continuous predictors were used as natural cubic spline functions [50]. The proportional hazards assumptions were checked using the Grambsch–Therneau method [24].

Similar analyses were performed for the secondary objectives DMFS, OS, and LRFS. The association between immunohistochemical ER, PR, Ki-67 expression (0–100%), and *FGFR1* amplification was also analyzed using summary statistics (median; interquartile range, IQR), box plots and Wilcoxon rank-sum tests. *P* values were not corrected for multiple testing.

All of the tests were two-sided, and a *P* value < 0.05 was regarded as statistically significant. Calculations were carried out using the R system for statistical computing (version 3.4.1; R Development Core Team, Vienna, Austria, 2017).

## Results

### Amplification of *FGFR1* in invasive breast cancer

#### *FGFR1* amplification rate in breast cancer

A total of 894 patients with breast cancer were initially included in the *FGFR1* analysis. Patients with contralateral breast cancer, breast cancer with distant metastasis at diagnosis, missing *FGFR1* data, and those without a positive observation time were excluded (*n* = 391; see Supplementary Table S1), resulting in a final sample size of 503 patients.

In this final cohort, amplification of the *FGFR1* gene was observed in 6.6% (33 of 503).

Missing tissue cores were the most common reason for nonassessable cases. Cases with no signals, or barely visible signals, were then excluded.

**Fig. 1:**
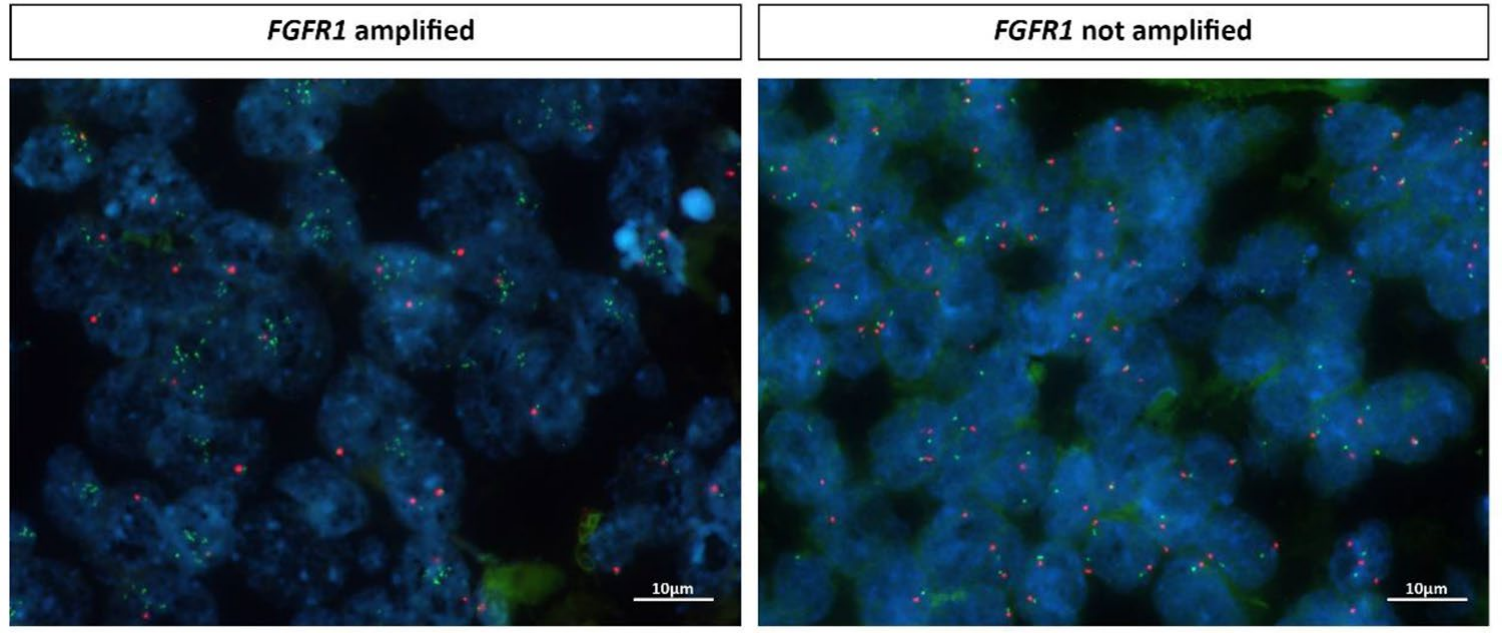
Illustration of *FGFR1* fluorescence in situ hybridization (FISH). Tumor nuclei are marked using DAPI, the *FGFR1* gene is depicted as green signal, the centromere (*CEN8*) is labeled with an orange signal (×1000, oil). One breast cancer case harbors *FGFR1* amplification, whereas the other breast tumor shows normal gene copy number of *FGFR1*, respectively

In Fig. 1, one breast cancer case with *FGFR1* amplification as well as one tumor without amplification but normal *FGFR1* gene status are illustrated.

#### Intratumoral homogeneity of *FGFR1* amplification

When comparing different intratumoral areas that were not lying close to each other, we did not find intratumoral heterogeneity of *FGFR1* amplification but perfect agreement regarding *FGFR1* gene status (TMA_1 vs. TMA2: agreement in 64 of 64 cases; TMA_1 vs. TMA_3: agreement in 42 of 42 cases; see Supplementary Table S2).

However, it has to be noted that 3 of 64 cases (4.7%, TMA_1 vs. TMA2) and 2 of 42 cases (4.8%, TMA_1 vs. TMA_3), respectively, harbored slightly increased *FGFR1* gene copy numbers with a *FGFR1*/*CEN8* ratio each that was very close to the defined cut-off (≥ 2.0), but did not surpass 1.99 (data not shown).

#### Association of *FGFR1* gene status and clinical and pathological parameters

The mean age of the patients with *FGFR1-*amplified breast cancer was 60 years, and they had a mean BMI of 27 kg/m^2^. More than half of these patients had pT1 tumors (54.5%) and 36.4% had positive lymph-node stages.

Most breast cancer patients with *FGFR1* amplification showed moderate differentiation (G2, 69.7%) and a luminal subtype with positive hormone receptor status and predominantly HER2- negative status (ER+, 90.9%; PR+, 78.8%; HER2+, 9.1%). The mean proliferation rate assessed using Ki-67 expression amounted to 24.8%. Table 1 lists the characteristics of the patients and tumors relative to *FGFR1* amplification.

**Table 1:** Patient and tumor characteristics relative to *FGFR1* amplification status

Characteristic	No *FGFR1* amplification (*n* = 470)		*FGFR1* amplification (*n* = 33)	
	Mean or *n*	SD or %	Mean or *n*	SD or %
Age (years)	57.5	12.7	60	10.4
BMI (kg/m^2^)	26	4.8	27	4.1
Ki-67 (%)	22.7	20.5	24.8	17.0
Tumor stage				
T1	261	55.5	18	54.5
T2	165	35.1	12	36.4
T3	22	4.7	2	6.1
T4	22	4.7	1	3.0
Lymph-node stage				
N0	287	61.1	21	63.6
N+	183	38.9	12	36.4
Grade				
G1	44	9.4	1	3.0
G2	312	66.4	23	69.7
G3	114	24.3	9	27.3
ER status				
ER−	100	21.3	3	9.1
ER+	370	78.7	30	90.9
PR status				
PR−	123	26.2	7	21.2
PR+	347	73.8	26	78.8
HER2 status				
HER2−	424	90.2	30	90.9
HER2+	46	9.8	3	9.1
Molecular subgroup				
TNBC	70	14.9	2	6.1
Luminal A-like	201	42.8	5	15.2
Luminal B-like	153	32.6	23	69.7
HER2-positive	46	9.8	3	9.1

*BMI* body mass index, *ER* estrogen receptor, *FGFR1* fibroblast growth factor receptor 1, *HER2* human epidermal growth factor receptor 2, *PR* progesterone receptor

Means and standard deviation (SD) are shown for continuous characteristics, frequency and percentage for categorical characteristics

**Fig. 2:**
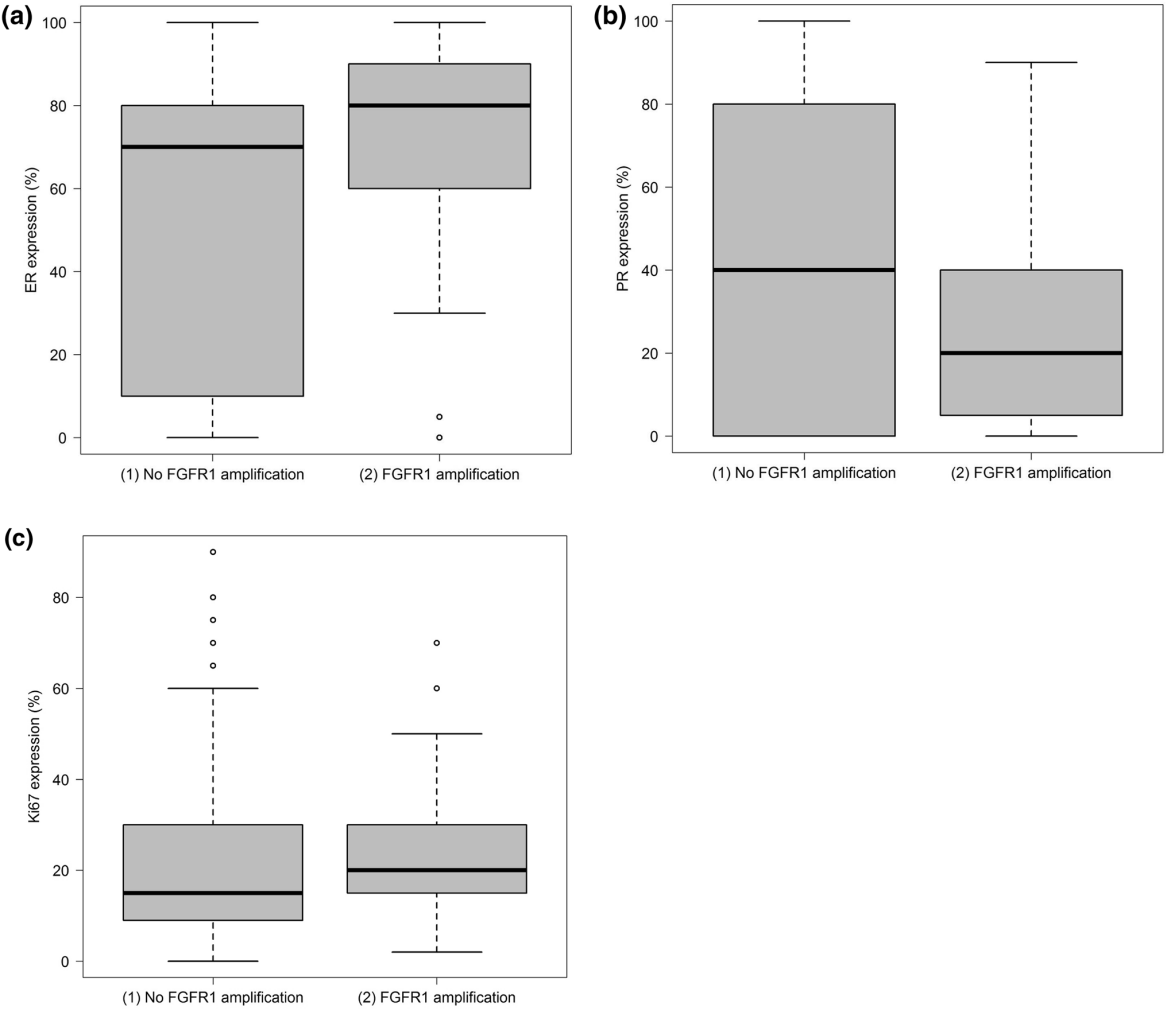
**a** Distribution of estrogen receptor (ER) expression relative to *FGFR1* amplification status (*P* < 0.01). **b** Distribution of progesterone receptor (PR) expression relative to *FGFR1* amplification status (*P* = 0.16). **c** Distribution of Ki-67 expression relative to *FGFR1* amplification status (*P* = 0.11)

Patients with breast cancer who had normal *FGFR1* copy numbers showed lower expression rate measurements (IHC) for the estrogen receptor (median, 70%; IQR, 10% to 80%) than patients with *FGFR1* amplification (median, 80%; IQR, 60% to 90%; Fig. 2a). Such associations were not seen for the progesterone receptor (Fig. 2b) or Ki-67 IHC (Fig. 2c).

There were no cases of increased copy numbers in more than one of the *FGFR* genes (i.e., *FGFR1* and *FGFR2* and/or *FGFR3* amplification).

Survival rates in *FGFR1*-amplified breast cancer.

#### Disease-free survival

The median follow-up period for the primary study aim of DFS was 10.0 years for patients both with and without *FGFR1* amplification. No significant differences were observed between breast cancer patients with and without *FGFR1* amplification in relation to DFS. The unadjusted HR was 1.60 (95% CI 0.88 to 2.89) and the adjusted HR was 1.25 (95% CI 0.67 to 2.32). The 5-year and 10-year survival rates are shown in Table 2.

**Table 2:** Numbers of events and survival rates for survival outcomes

Survival outcome	Patient group	At risk	Events	5-year survival rate (95% CI)	10-year survival rate (95% CI)
Disease-free survival	No *FGFR1* amplification	470	124	0.85 (0.82, 0.88)	0.72 (0.68, 0.77)
	*FGFR1* amplification	33	12	0.72 (0.59, 0.90)	0.62 (0.47, 0.82)
Overall survival	No *FGFR1* amplification	470	83	0.92 (0.89, 0.94)	0.81 (0.78, 0.85)
	*FGFR1* amplification	33	9	0.82 (0.69, 0.96)	0.71 (0.57, 0.89)
Distant disease-free survival	No *FGFR1* amplification	470	66	0.90 (0.87, 0.93)	0.82 (0.78, 0.86)
	*FGFR1* amplification	33	8	0.75 (0.61, 0.93)	0.70 (0.55, 0.90)
Local recurrence-free survival	No *FGFR1* amplification	470	38	0.95 (0.93, 0.97)	0.89 (0.86, 0.93)
	*FGFR1* amplification	33	5	0.84 (0.71, 1.00)	0.79 (0.64, 0.98)

*CI* confidence interval(s), *FGFR1* fibroblast growth factor receptor 1

#### Distant metastasis-free survival, overall survival, and local recurrence-free survival

**Fig. 3:**
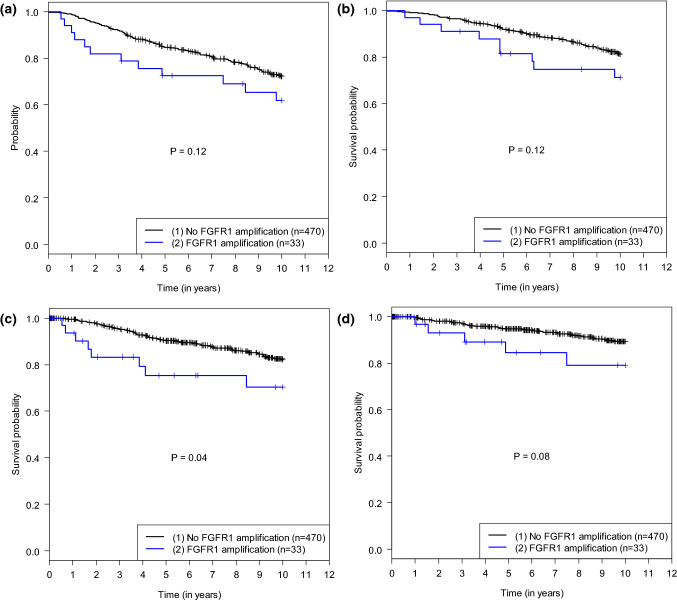
**a** Kaplan–Meier curves for disease-free survival (DFS) relative to *FGFR1* amplification status with log-rank test *P* value. **b** Kaplan–Meier curves for overall survival (OS) relative to *FGFR1* amplification status with log-rank test *P* value. **c** Kaplan–Meier curves for distant metastasis-free survival (DMFS) relative to *FGFR1* amplification status with log-rank test *P* value. **d** Kaplan–Meier curves for local recurrence-free survival (LRFS) relative to *FGFR1* amplification status with log-rank test *P* value

Breast cancer patients with *FGFR1* amplification had a poorer DMFS than patients without amplification (*P* = 0.04, unadjusted analysis); however, this difference in DMFS outcome did not achieve significance in the adjusted analysis (*P* = 0.05). No significant impact of *FGFR1* amplification on the other secondary outcomes, OS and LRFS, was observed. Survival rates and HRs are presented in Tables 2 and 3. Kaplan–Meier curves are shown in Fig. 3.

**Table 3:** Unadjusted and adjusted hazard ratios for *FGFR1* amplification versus nonamplification

Survival outcome	Unadjusted HR (95% CI)	*P* value	Adjusted HR^a^ (95% CI)	*P* value
Disease-free survival	1.60 (0.88, 2.89)	0.12	1.25 (0.67, 2.32)	0.48
Overall survival	1.72 (0.87, 3.43)	0.12	1.18 (0.56, 2.47)	0.66
Distant disease-free survival	2.15 (1.03, 4.48)	0.04	2.08 (0.98, 4.39)	0.05
Local recurrence-free survival	2.25 (0.88, 5.71)	0.09	2.29 (0.89, 5.91)	0.09

*CI* confidence interval(s), *HR* hazard ratio

^a^Hazard ratios were adjusted for age at diagnosis, body mass index, tumor stage, tumor grade, lymph-node stage, estrogen receptor status, progesterone receptor status, HER2 status, and Ki-67

### *FGFR2* and *FGFR3* amplification in breast cancer

The evaluation of *FGFR2* and *FGFR3* gene status only revealed a very low frequency of copy number aberrations for each gene in the FISH analysis. *FGFR2* was amplified in 0.9% (four of 447 cases assessable using *FGFR2* FISH); only one case harbored *FGFR3* amplification (0.2%, one of 562 cases assessable using *FGFR3* FISH). Due to the low numbers of amplified cases, *FGFR2* and *FGFR3* gene status was excluded from further survival analysis.

## Discussion

This study investigated the amplification rates of the fibroblast growth factor receptor genes *FGFR1*, *FGFR2,* and *FGFR3* in patients with breast cancer. In view of the very small numbers of *FGFR2-*amplified and *FGFR3*-amplified cases, the analyses were on outcomes in patients with *FGFR1* amplification. Amplification of *FGFR1* was seen in 6.6% of assessable BC cases. Earlier studies have reported higher amplification rates of *FGFR1* and the corresponding chromosomal region 8p11-12 (8.7–13.2%) [7, 13, 19, 54]. The fact that the frequency of *FGFR1* amplification in the present study was lower might be due to different methods of evaluating gene status (e.g., multiplex ligation-dependent probe amplification), a different composition of the cohort (e.g., varying distribution of intrinsic subtypes), and the fact that the *FGFR1* gene was not always included in the amplification unit in the earlier studies. The present study did not investigate variations in gene copy numbers for other genes included in the previously described 8p11.2-p12 amplicon [9].

In this study, BC patients with *FGFR1* amplification showed a trend toward poorer outcomes, especially DMFS and LRFS. *FGFR1* amplification was not an independent predictor of shorter DFS or OS. Thus, the study does not fully confirm the findings of an earlier report that *FGFR1*-amplified BC was associated with poorer OS in the overall cohort and that *FGFR1* amplification was predictive of poor DFS, OS, and DMFS in ER-positive patients with BC [13]. Cuny et al. reported a shorter DFS in *FGFR1-*amplified BC in comparison with nonamplified carcinomas. Intriguingly, co-amplification of *FGFR1* and the cyclin D1 gene (*CCND1*) showed even poorer DFS than increased *FGFR1* copy numbers without *CCND1* amplification [8]. It may be presumed that varying distributions of intrinsic subtypes contribute to these different findings. It should also be mentioned that the survival analyses in the present study were limited, as the cohort investigated included only 33 cases of *FGFR1-*amplified BC. In another study, *FGFR1* amplification was not associated with relapse-free survival (RFS) or BC-specific survival. Instead, protein expression predicted shorter RFS in ER-positive/HER2-negative BC [56]. Further comprehensive studies are therefore needed in order to investigate the impact of *FGFR1* amplification on survival in BC patients.

Estrogen receptor-positive BC [7] and luminal B BC [61] have been reported to show the highest frequency of *FGFR1* amplifications. In one study, the amplification rates were 21.0% for luminal B, 12.7% for basal-like, 10.4% for luminal A, and 7.1% for HER2-positive invasive BC [29]. Interestingly, luminal A BC that expressed high levels of *FGFR1* has been found to behave more aggressively, with a prognosis similar to that in luminal B tumors [51]. In the present study, most *FGFR1*-amplified tumors were ER-positive (90.9%), a finding that is consistent with the results published by Moelans et al. [39]. The present study showed that 69.7% of BCs with *FGFR1* amplification harbored a luminal B phenotype, whereas the other intrinsic subtypes were only found at lower frequencies, up to 15.2%. However, due to the small numbers of *FGFR1*-amplified cases in the study, survival analysis of intrinsic subtypes relative to *FGFR1* copy number status was not feasible.

Inhibition of *FGFR1* has been regarded as a potential therapeutic target. On the assumption that the receptor tyrosine kinase (RTK) *FGFR1* is the driver of the 8p11.2-p12 amplicon and represents a potential drug target in a variety of cancers, RTK-targeting small-molecule inhibitors against *FGFR1* such as ponatinib, dovitinib, PD173074, and SU5402 were designed, and knockdown and preclinical pharmaceutical inhibition studies were carried out [2, 5, 10, 23, 31, 47, 62]. Multikinase inhibitors (TKIs), such as lucitanib (E-3810, NCT01283945), dovitinib (TKI258), nintedanib, and ponatinib [42], and selective *FGFR* inhibitors, such as AZD4547 [18] (NCT00979134, NCT01202591), BGJ398 (NCT01004224), LY2874455 (NCT01212107), and JNJ-42756493 (NCT01703481), have been tested in several phase I and II trials in cancers with *FGFR1* aberrations [10]. In patients with advanced BC, the overall response rate was up to 50% and progression-free survival up to 10.9 months when the agents were combined with fulvestrant, but overall, the desired efficacy of FGFR inhibitors has not been achieved in (pre-)clinical studies [41, 42]. Administration of multi-TKIs was accompanied by asthenia, gastrointestinal symptoms, hypertension, and lymphopenia, whereas selective FGFR inhibitors led to hyperphosphatemia, gastrointestinal symptoms, nail toxicity, and stomatitis [2, 42]. Amplification of *FGFR1* was seen in up to 43% of patients with invasive lobular cancer (ILC) of the breast, and it was associated with expression. *FGFR1* inhibition has been found to reduce the viability of the BC cell line MDA-MB-134, which has similarities to ILC in relation to some copy number variations (including *FGFR1*) and protein expression [49]. In *FGFR1*-amplified ILC, ribosomal S6 kinase (RSK) inhibitors may be another potential drug target, as Xian et al. observed an effect of RSK on *FGFR1*-transformed cells [68]. In addition, simultaneous inhibition of *FGFR1* and vascular endothelial growth factor receptor 1 (VEGFR1) may lead to anti-angiogenic effects in vivo [21]. ODM-203 acts as a selective dual blockade of FGFR and VEGFR, but may cause hyperphosphatemia and bilirubinemia [27]. There is evidence that high levels of *FGFR1* expression are associated with resistance to anti-HER2 therapy in patients with HER2-positive BC [25]. It needs to be tested whether a combination of anti-HER2 therapy and FGFR inhibition might help to resolve this issue. In addition, FGFR inhibition may reverse resistance to endocrine therapy and anti-CDK4/6 therapy, and since 26.4% of *FGFR1*-amplified BC has been found to have *PIK3CA* alterations [26], it may be combined with inhibitors of the PI3K pathway. However, further investigation of the efficacy and safety of the inhibitors and combinations of these agents is needed [42].

*FGFR1* amplification is associated with increased expression [33, 61]. However, it needs to be borne in mind that increased copy numbers may not always predict high levels of FGFR1 protein expression [48], so that inhibition might fail if treatment were to be selected on the basis of *FGFR1* amplification. This may be related to the quality of the FISH assay and IHC assay used. Measurement of protein or mRNA expression may lead to better prediction of the response to FGFR inhibitors in head and neck cancer [20], but this issue has yet to be investigated in BC [42]. One limitation of our study is that, up to date, we did not analyze the association between *FGFR1* amplification and protein expression of FGFR1. Further investigations have to show whether the copy number gain of the gene lead to increased expression of this growth factor receptor in our breast cancer cohort and whether FGFR1 overexpression might predict prognosis.

The 8p11-12 amplicon was significantly associated with DFS and distal recurrence [6]. However, it is still controversial whether *FGFR1* itself is the driver oncogene, or whether another gene in the 8p11.2-p12 amplicon is responsible for the oncogenic potential [7, 13, 19]. *FGFR1* amplification is apparently not always associated with *FGFR1* overexpression, and the oncogenic amplicon 8p11.2-p12 is not always accompanied by *FGFR1* amplification and sensitivity to FGFR inhibition [48, 55]. It therefore remains unclear whether *FGFR1* is the appropriate drug target, or another gene in the amplicon, and this has led to ongoing discussion and investigation of this issue.

In addition to *FGFR1* copy number aberrations, an *FGFR1* SNP (rs17182023) has been investigated and was found to be associated with a reduced risk of BC and lower *FGFR1* expression. By contrast, high levels of *FGFR1* were associated with a poor outcome [67].

The *FGFR2* locus has been shown to be one of the regions associated most strongly with the risk of BC in genome-wide association studies [12, 15, 35–37]. Rather than increased gene copy numbers, SNPs in the *FGFR2* risk locus appear to be associated with the development of BC. Campbell et al. reported reduced *FGFR2* expression and consequently—due to less influence of *FGFR2* on the estrogen regulon—increased responsiveness to estrogen if one of three *FGFR2* variants existed [3]. Copy number aberrations appear to be less important in BC. The *FGFR2* gene was amplified in only 0.9% of the patients with BC in the present study, which is lower than the rate of 4.4% described in the literature [7]. This might be due to the composition of the cohort, since Turner et al. reported *FGFR2* amplification in 4.0% of triple-negative BCs, but not in any other subtype [60]. In view of the poor prognosis for patients with TNBC without treatment and the current lack of an approved targeted therapy, FGFR inhibitors may be a treatment option in *FGFR2*-amplified/*FGFR2*-overexpressing TNBC. Initial preclinical data are promising [58], but this has yet to be confirmed in clinical studies.

The receptor tyrosine kinase *FGFR3* may influence hormone receptor-positive BC that is resistant to tamoxifen [57]. However, amplification of *FGFR3* is fairly exceptional in BC, as it was found in 0.2% of the present cohort and in 0.8% of BC cases investigated by Helsten et al. [26]. The SNP *FGFR3*_rs743682 was found to be associated with the risk of BC, but did not reach the same association level as the SNP *FGFR2*_ rs2981582 [1].

It is not only copy number variations and SNPs in the *FGFR* genes that can lead to high expression of the respective fibroblast growth factor receptor and up-regulated FGFR pathways. Mutations, rearrangements, post-transcriptional regulation, and isoform switching/alternative splicing, as well as stimulation via fibroblast growth factors from tumor or stromal cells, can also have an impact on the system [62]. For instance, the *FGFR1* splice variant IIIb has been reported to inhibit cell growth [32]. These numerous changes should be borne in mind when FGFR-targeted therapy is being tested.

In conclusion, fibroblast growth factor receptor alterations (e.g., *FGFR1* copy number variations and *FGFR2* SNPs) influence the risk and prognosis in patients with breast cancer. Further investigations of the dysregulated FGFR pathways and the effects of FGFR inhibitors are needed. Due to the complexity of the *FGFR1* amplicon itself, and because of the results that have been published so far, clarification of the driver gene of the *FGFR1* amplicon 8p11.2-p12 is warranted in order to identify the potential target gene.

## Electronic supplementary material

Below is the link to the electronic supplementary material.

Supplementary file1 (DOCX 38 kb)

## Abbreviations

BBCC: Bavarian Breast Cancer Cases and Control (study)
BC: Breast cancer
BMI: Body mass index
CI: Confidence interval(s)
DFS: Disease-free survival
DMFS: Distant metastasis-free survival
ER: Estrogen receptor
FGFR: Fibroblast growth factor receptor
FISH: Fluorescence in situ hybridization
HER2: Human epidermal growth factor receptor 2
HR: Hazard ratio
IHC: Immunohistochemistry
ILC: Invasive lobular cancer
IQR: Interquartile range
LRFS: Local recurrence-free survival
OS: Overall survival
PR: Progesterone receptor
RFS: Relapse-free survival
RSK: Ribosomal S6 kinase
RTK: Receptor tyrosine kinase
TKI: Multikinase inhibitor
TMA: Tissue microarray
VEGFR1: Vascular endothelial growth factor receptor 1
