# Supplementary material for: Impact of fibroblast growth factor receptor 1 (FGFR1) amplification on the prognosis of breast cancer patients
Source: Breast Cancer Res Treat. 2020 Aug 27;184(2):311–24. doi: 10.1007/s10549-020-05865-2 (PMC7599145; doi:10.1007/s10549-020-05865-2)
Supplement: Supplementary file 1 — Supplementary file1 (DOCX 38 kb) [file 10549_2020_5865_MOESM1_ESM.docx]

**Supplementary material**

**Supplementary Table S1** Exclusion of patients.

|  | Excluded (*n*) | *n* |
| --- | --- | --- |
| All patients with breast cancer | – | 894 |
| Patients with contralateral breast cancer before or simultaneous with ipsilateral breast cancer | 48 | 846 |
| Patients without *FGFR1* gene status information | 320 | 526 |
| Patients with distant metastases at diagnosis | 22 | 504 |
| Patients without positive observation period | 1 | 503 |

*FGFR1,* fibroblast growth factor receptor 1.

**Supplementary Table S2** Exclusion of intratumoral heterogeneity of *FGFR1* amplification. When comparing three different intratumoral areas that were not lying close to each other (Table S2a: TMA_1 vs. TMA2 and Table S2b: TMA_1 vs. TMA_3), there was no significant intratumoral heterogeneity of *FGFR1* amplification, but perfect agreement regarding *FGFR1* gene status (amplification defined by a *FGFR1*/*CEN8* ratio >2.0 vs. no amplification). However, it has to be noted that 3/64 (TMA_1 vs. TMA2) and 2/42 cases (TMA_1 vs. TMA_3), respectively, harboured slightly increased *FGFR1* gene copy number with a *FGFR1*/*CEN8* ratio that was very close to the defined cut-off (>2.0), but did not surpass 1.99 (data not shown).

| Table S2a | | TMA_2 (*n*=64) | |
| --- | --- | --- | --- |
|  |  | not amplified | amplified |
| TMA_1 | not amplified | 59 | 0 |
|  | amplified | 0 | 5 |

| Table S2b | | TMA_3 (*n*=42) | |
| --- | --- | --- | --- |
|  |  | not amplified | amplified |
| TMA_1 | not amplified | 38 | 0 |
|  | amplified | 0 | 4 |
